# Perinatal management and follow-up in a child with a prenatal diagnosis of OTC deficiency: a case report

**DOI:** 10.3389/fnut.2024.1416466

**Published:** 2024-09-30

**Authors:** Álvaro Martín-Rivada, Mercedes Murray Hurtado, Elena Martín-Hernández

**Affiliations:** ^1^Servicio Pediatría, Sección de Nutrición y Errores Innatos del Metabolismo, Complejo Hospitalario Universitario de Canarias, San Cristóbal de La Laguna, Spain; ^2^Centro de Referencia Nacional (CSUR) y Europeo (MetabERN) en Enfermedades Metabólicas, Hospital Universitario 12 de Octubre, Instituto de Investigación i+12, CIBERER, Madrid, Spain

**Keywords:** urea cycle disorders, ornithine transacarbamylase deficiency, hyperammonemia, glycerol phenylbutyrate, inborn errors metabolism

## Abstract

Ornithine transcarbamylase deficiency (OTCD) is the most common disorder of the urea cycle and is caused by a mutation of the *OTC* gene, located on chromosome X. Its prevalence is estimated at 1 in 80,000 to 56,500 births, but this X-chromosomal inheritance results in males being more affected than females. In neonates affected with this disorder, hyperammonemia after birth can lead to neurological and liver damage that can be fatal. We present a child with a prenatal diagnosis based on an older sibling with the same pathology, which led us to adopt an intensive treatment since the delivery. He was admitted in a neonatal unit and treatment with protein restriction, 10% glucose saline serum and glycerol phenylbutyrate was initiated. To date, after 3.5 years of follow up, growth and neurological development have been adequate, biochemical control has been appropriate except for a simple and mild decompensation during the course of a gastroenteritis. This case emphasises the importance of early diagnosis and treatment to avoid potential complications.

## Introduction

Ornithine transcarbamylase deficiency (OTCD) is the most common disorder of the urea cycle and is caused by mutations in the *OTC* gene (OTC; OMIM 300461), located on chromosome Xp11.4 (1). The urea cycle is the process by which ammonium, a neurotoxic substance formed because of protein metabolism, is converted into urea, which is excreted (2). OTCD is the only X-linked urea cycle disorder, and its prevalence is estimated at 1 in 80,000 to 56,500 births (3). This X-chromosomal inheritance results in males being more affected than females. Males with severe neonatal-onset OTC deficiency may become symptomatic in the first week of life, frequently on days two to three of life, with a catastrophic onset by the time they come to medical attention (4). Males and heterozygous females with post-neonatal-onset (partial) OTC deficiency can present from infancy to later childhood, adolescence, or adulthood (1).

The prognosis in children with urea cycle disorders (UCDs) presenting in the neonatal period is generally poor; half of the children who survive neonatal-onset OTCD die before entering school and those who survive have a high incidence of developmental disabilities (5), so early diagnosis and treatment is essential as it can improve survival and avoid cognitive impairment (6, 7). Therapeutic principles for neonatal acute hyperammonemia in the management of urea cycle disorders are based on avoiding catabolism, protein restriction and the use of intravenous drugs for nitrogen scavenging (8). Knowledge on long-term management is still poor, beyond dietary optimisation and pharmacological treatment (9), however, so far, the goal of preventing hyperammonemia has proven useful to achieve a correct development with a good quality of life (1, 10, 11).

Herein, we report perinatal management considerations and the follow up to the age of 3 years of a male patient in whom prenatal diagnosis of the disease could be performed because he had an older sibling affected.

## Case presentation

His older brother, born in 2014, was the index case. At 13 months, he was diagnosed with OTCD due to a hyperammonemic episode (300 μmol/L), presented with encephalopathy and hepatopathy. Growth failure was already noted at diagnosis. Genetic study was performed revealing that he carried the c.119G>A, p.Arg40His mutation in the *OTC* gene, classified as pathogenic. The segregation study showed that the mother was also a carrier. Father was healthy. Treatment with protein restriction, citrulline supplementation and ammonium scavenger was initiated: sodium phenylbutyrate until 2019 and glycerolphenylbutyrate (GPB) thereafter. He developed several nutrition deficiencies: long-chain polyunsaturated fatty acids (LC-PUFA), selenium, carnitine and zinc. To date, he has suffered 5 moderate hyperammonemic decompensations (ammonium levels: 150–300 μmol/L). Psychomotor and growth development are currently normal.

Patient’s mother became pregnant with the younger brother at the age of 32. At that time, she had multiple sclerosis, hypothyroidism and partial OTCD on treatment with carnitine, citrulline, coenzyme Q10 and vitamins, without protein restriction. No episodes of hyperammonemia have been observed. Glutamine levels were in the normal range. Several attempts of *in-vitro* fertilisation with embryo selection were performed but remained unsuccessful and she became spontaneously pregnant thereafter.

Throughout the pregnancy, the mother was closely monitored. Serial ammonium and amino acid levels were always normal, and she did not show any decompensation. An amniocentesis was performed showing that the foetus carried the p.Arg40His mutation in the *OTC* gene. At 37 weeks of gestation, labour was induced by vaginal delivery without complications. Early epidural anaesthesia was administered, and she received treatment with 10% glucose saline serum, ondansetron and carnitine. The newborn was born asymptomatic (Apgar 9/9). Neonatal anthropometry was as follows, according to WHO standards 2005/2006 (12): weight 3,050 g (p50–p75), height 50 cm (p75–p90), and head circumference 34.5 cm (p50–p75). He was admitted to the neonatal intensive care unit, during the first hour of life, perfusion with 10% glucose saline (4 mL/kg/h) was started together with oral treatment with GPB (initial dose: 8.5 mL/m^2^/day) and citrulline (170 mg/kg/day). After 4 h, enteral nutrition was initiated, with protein-free formula and, in the following days, protein intake was progressively increased from 0.5 to 1.6 g/kg/day (mixed breast milk and formula with essential amino acids) with a progressive decrease in intravenous glucose intake, until its suspension on the third day of life. The neonate remained asymptomatic all the time, serial biochemical controls with ammonium levels were performed and found to be normal. After 1 week, he was discharged from the hospital to continue outpatient follow-up.

**Figure 1:**
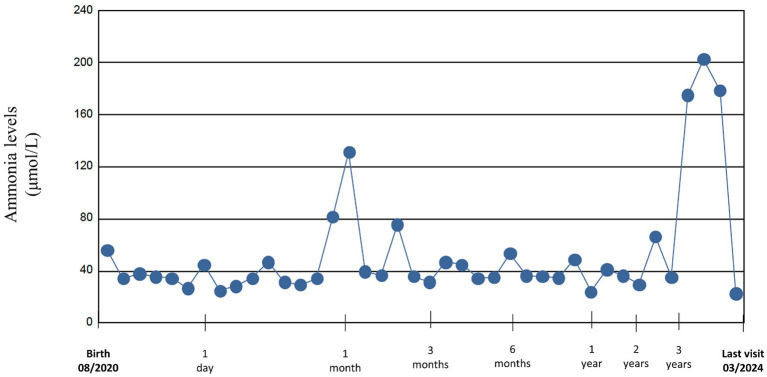
Ammonium levels from birth to last visit.

**Figure 2:**
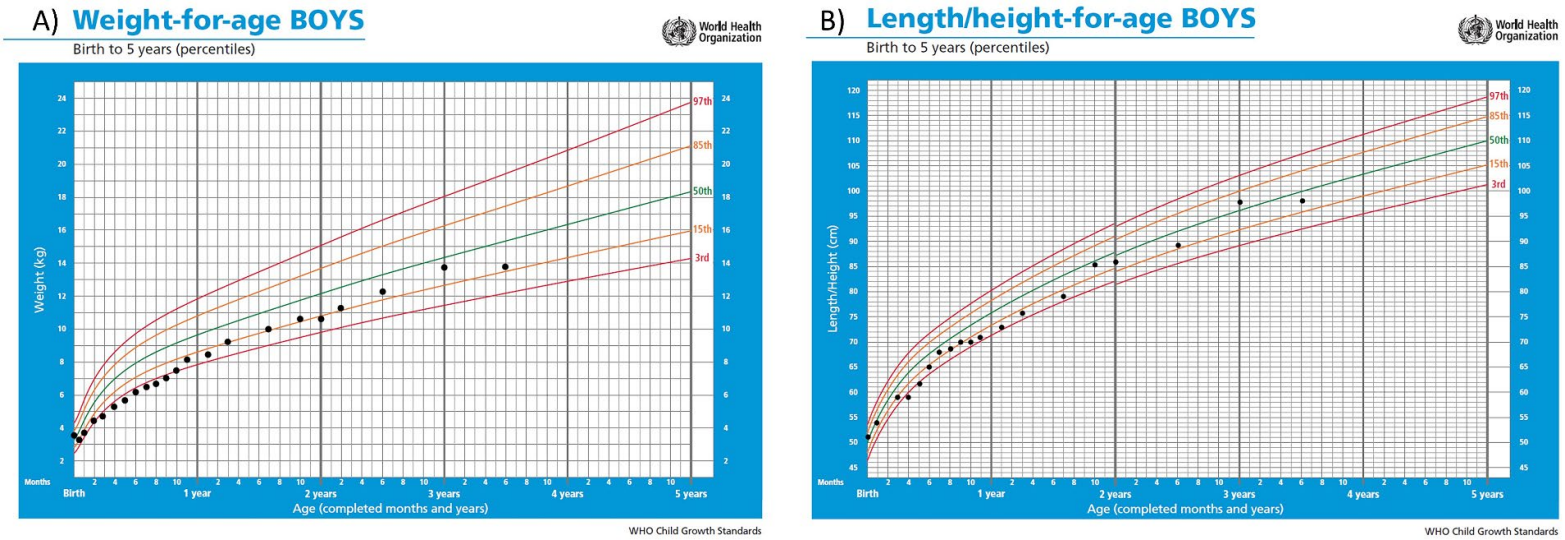
Curves of weight **(A)** and height **(B)** percentiles according to WHO standards.

During the first months of life protein intake was reduced up to 1 g/kg/day due to fluctuating and slightly elevated ammonium levels (up to 81 μmol/L) (Figure 1). The patient remained asymptomatic throughout, with no clinical decompensation. A drop in weight and height percentiles was observed (Figure 2). Biochemical control together with therapeutic data is shown in Table 1. Clinical and biochemical follow-up was closer during the first year of life, according to the Spanish protocol (13), but only the most relevant data are shown herein. During the entire follow-up controlled protein diet supplemented with essential amino acids was maintained, together with citrulline (120–200 mg/kg/day) and GPB treatment (350–500 mg/kg/day). Clinical and biochemical controls including urinary phenylacetylglutamine (PAG) were performed at least every 6 months and treatment was adjusted if necessary. Clinical evolution has been satisfactory. He has suffered only one mild decompensation, in November 2023, at the age of 3 years, during an episode of gastroenteritis with vomiting and absence of oral tolerance, where the presence of moderate hyperammonemia (200 μmol/L) was observed, which resolved briefly within 2 h with the use of intravenous arginine and sodium benzoate.

**Table 1:** Biochemical and therapeutic data in a child with a prenatal diagnosis of OTC deficiency.

	1 m	3 m	6 m	12 m	1.5 y	2 y	2.5 y	3 y	3.5 y
Biochemical data									
Ammonia (μmol/L) (N.V: <50)	121	32	53	24	36.29	29.7	66.35	35	22.8
Glutamine (μmol/L) (N.V: 350–650)	1066.3	369.5	819.0	1035.6	992	851.5	1,387	854.8	621.9
Leucine (μmol/L) (N.V: 50–150)	123.7	46.4	50.1	120.7	155	49.24	78.98	60.3	65.2
Valine (μmol/L) (N.V: 130–300)	193.9	82.0	105.0	176.4	238.1	101.7	149.9	87	148.2
Isoleucine (μmol/L) (N.V: 30–850)	62.8	25.6	27.8	56.0	90.0	27.66	34.18	33.8	36.6
Arginine (μmol/L) (N.V: 30–150)	21.7	28.9	23.7	59.3	35.4	64.56	24.18	29.7	40
PAG (mcg/mL)	N.D	3,697	13,745	27,564	N.D	35,516	3018.7	41,941	N.D
Therapeutic data									
Total protein intake (g/kg/day)	1.33	1.2	1.0	1.2	1.2	1.3	1.15	1.1	1.2
Citrulline (mg/kg/day)	182	170	147	145	188	176	160	184	200
GPB (mg/kg/day)	468	453	503	396	500	442	412	470	460

GPB, glycerol phenylbutirate; m, months; N.D, no data; N.V, normal values; PAG, phenylacetylglutamine; y, years.

**Figure 3:**
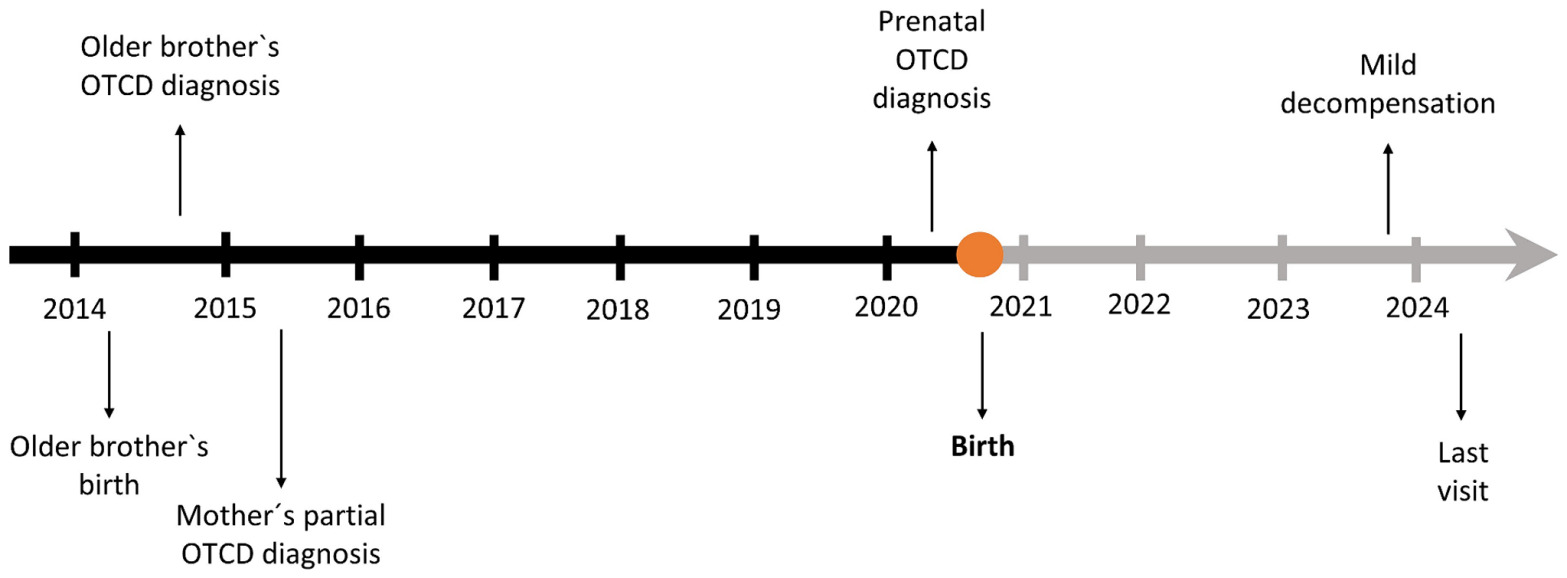
Timeline of the case.

In March 2024, at the age of 3.5 years, last clinical visit was performed. Current treatment is GPB 460 mg/kg/day, citrulline 200 mg/kg/day and a total protein intake of 1.1 g/kg/day (34% as protein equivalent essential amino acids). In the examination anthropometry was as follows: weight 13.8 kg (P19, −0.87DE), height 98 cm (P31, −0.49DE), BMI 14.37 kg/m^2^ (P18, −0.90DE), showing an adequate growth and normal psychomotor development. He currently attends a regular education school with good performance and meets age-appropriate motor and intellectual development milestones amino acids and ammonium levels remained within the normal range. The timeline of the case report is shown in Figure 3.

## Discussion

Here, we describe the case of a newborn with a prenatal diagnosis of OTC deficiency in the clinical setting of a sibling with the same disease, diagnosed approximately at 1 year of life and a mother with partial deficiency. For her next pregnancy, the mother underwent unsuccessful *in-vitro* fertilisation with oocyte selection, a technique that allows the preselection of an OTC-free embryo to prevent subsequent risks (14). Finally, she became spontaneously pregnant, and an amniocentesis was performed showing that the foetus was affected. As this disorder can lead to hyperammonemia after birth, causing symptoms that, although at first may seem unspecific (refusal of food, vomiting, and lethargy), eventually lead to coma and even premature death (4), early detection and rapid management of these patients is essential for their survival. Additionally, female carriers are at risk of pregnancy complications too, as both the intrapartum and postpartum periods are catabolic states, possibly triggering hyperammonemia, which can cause life-threatening encephalopathy (15, 16). Therefore, a multidisciplinary and coordinated plan of action was implemented for the delivery, according to the existing recommendations (17, 18).

Concerning the therapeutic approach of the newborn we consulted the British Inherited Metabolic Disease Group (BIMDG) guidelines, the international guidelines and the previous data about the p.Arg40His mutation. According to the guidelines, if the previous sibling had become ill after the neonatal period, a more conservative approach could be adopted, initiating treatment with clinical and biochemical follow-up to monitor progress (10, 19). On the other hand, the p.Arg40His has been previously reported in several cases (20). Although it was initially described to confer a late onset type of presentation (21) but in a large cohort it was detected in patients with an onset from the first month of life (22). However, in this case, given the severity of the sibling’s debut episode and the evolution with several decompensations, a more proactive approach was decided, with intensive treatment from the time of birth, based on high intravenous glucose intake and initiation with GBP in the first hour of life, and a first dose of protein-free formula if he remained asymptomatic after 4 h (10). GBP was chosen based on the experience in the sibling’s case, and the recent evidence, which reflects the efficacy and safety of its use in the paediatric population (23) and in neonates (24).

During the outpatient follow-up of the first months, close management was necessary to find a balance between sufficient protein intake to allow adequate growth but without episodes of hyperammonemia. In the first months of age there was a decrease in weight and length percentiles that could be related to protein restriction. In fact, growth is frequently affected in individuals with urea cycle disorders (11). However, it is true that his older brother showed a similar growth pattern during the first year of life, prior to diagnosis and subsequent protein restriction, which could be indicative of a certain component of familial growth pattern. It has been reported that asymptomatic patients without hyperammonemia in the early stages of life can achieve normal weight and height curves (11, 25).

Treatment with citrulline and GPB was adjusted according to growth and biochemical data. Urinary PAG levels were also monitored, as they have shown to be a useful biomarker in patients with urea cycle disorders on GPB therapy (26, 27).

Ammonium levels remained within the normal range except for two episodes of hyperammonemia at 1 month and 3.5 years of age, as shown in Figure 2. In the first months, fluctuating values with slight elevations were seen, where therapeutic adjustments were performed. However, there were also difficulties in obtaining venous samples, so it is unclear whether these fluctuations were due to this setback or to suboptimal management. The other event was the episode of gastroenteritis with mild hyperammonemia, which resolved briefly, uneventfully and with no apparent sequels.

Another aspect that remains to be fully elucidated in these pathologies is the performance and timing of liver transplantation. Despite evolving medical treatments, patients with urea cycle disorders remain at high risk of hyperammonemia, so liver transplantation has also been performed as an alternative. These have the potential to “cure” the pathology, but it is a surgical procedure that involves risks of mortality and morbidity, as well as requiring a lifelong immunosuppression regimen (28, 29). However, as different gene therapy modalities are emerging, including adeno-associated virus gene addition, mRNA therapy and genome editing (30), the indications for liver transplantation will have to be revised.

In summary, this case demonstrates the complexity in the management of a neonate with prenatal OTCD diagnosis where treatment with protein restriction and ammonium scavenger was started at birth. However, the aggressiveness of the strategy at the time of delivery when a prenatal diagnosis is available, the strictness of nutritional control during the first months of life, and the ideal time at which liver transplantation or novel therapies could be considered in patients with this profile, are still aspects to be discussed. Therefore, further studies and other reported cases will be needed in the future to build stronger evidence for optimising the management of patients with prenatal diagnosis of this urea cycle disorder.

## Data Availability

The original contributions presented in the study are included in the article/supplementary material, further inquiries can be directed to the corresponding author.
